# Supplementary material for: A dual-labeling probe to track functional mitochondria–lysosome interactions in live cells
Source: Nat Commun. 2020 Dec 8;11:6290. doi: 10.1038/s41467-020-20067-6 (PMC7722883; doi:10.1038/s41467-020-20067-6)
Supplement: Supplementary file 1 — Supplementary Information [file 41467_2020_20067_MOESM1_ESM.pdf]

Supplementary Materials

A dual-labeling probe to track functional mitochondria–lysosome interactions in live cells

Qixin Chen, Hongbao Fang, Xintian Shao, Zhiqi Tian, Shanshan Geng, Yuming Zhang, Huaxun Fan, Pan Xiang, Jie Zhang, Xiaohe Tian, Kai Zhang, Weijiang He, Zijian Guo, Jiajie Diao

Supplementary Figure 1. Synthetic scheme of Coupa
Supplementary Figure 2. ^{1}H NMR spectra of Coupa in CD_3OD.
Supplementary Figure 3. ^{13}C NMR of Coupa in CD_3OD.
Supplementary Figure 4. High-resolution mass spectrometrum of Coupa.
Supplementary Figure 5. Fluorescent spectra of Coupa determined under different conditions.
Supplementary Figure 6. Co-localization coefficients of MTG-labeled mitochondria with the Coupa-labeled blue and red fluorescent particles.
Supplementary Figure 7. SIM imaging of Mito-V-labeled mitochondria in HeLa cells with or without CCCP treatment.
Supplementary Figure 8. Normalized mean fluorescence intensity of Coupa-labeled blue florescent particles in untreated and CCCP-treated HeLa cells.
Supplementary Figure 9. SIM imaging of CCCP-treated HeLa cells co-stained by Coupa and GFP via a coupa-mito/GFP dual channel mode.
Supplementary Figure 10. SIM imaging of HeLa cells co-stained by Coupa and mitochondria-GFP with or without CCCP treatment.
Supplementary Figure 11. Overlap SIM images of Coupa-labeled red particles and lysosome-tracker-green (LTG) labeled lysosomes in untreated HeLa cells.
Supplementary Figure 12. Characterization of Coupa-labeled red florescent particles in living HeLa cells.
Supplementary Figure 13. Investigation of the cell uptake of Coupa by HeLa cells via SIM imaging.
Supplementary Figure 14. Photobleaching comparison of DAPG and Coupa-lyso fluorescence in living HeLa cells.
Supplementary Figure 15. Co-localization between lysosomes and autolysosomes in CCCP-treated HeLa cells stained by Coupa and LTR via SIM imaging.
Supplementary Figure 16. Fluorescence lifetime imaging of Coupa-mito labeling mitochondria in live HeLa cells.
Supplementary Figure 17. The normalized mean intensity of Coupa-mito-stained and MTG-stained mitochondria in CCCP-treated HeLa cells.
Supplementary Figure 18. The cristae-to-cristae distance determination in HeLa cells via SIM imaging.
Supplementary Figure 19. Fluorescence recovery after photobleaching assay (FRAP) for Coupa-mito and MTG in HeLa cells stained by both Coupa and MTG.
Supplementary Figure 20. The original trajectory for detection the Coupa-mito and MTG fluorescence intensity shown in Figure 4c.

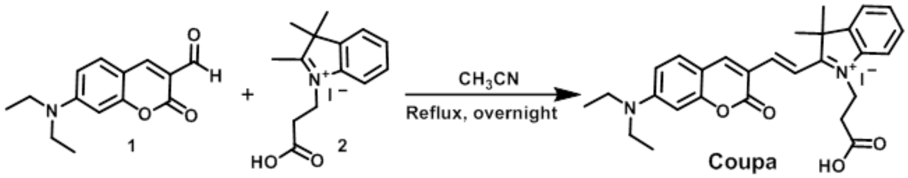

Supplementary Figure 1. Synthetic scheme of Coupa.

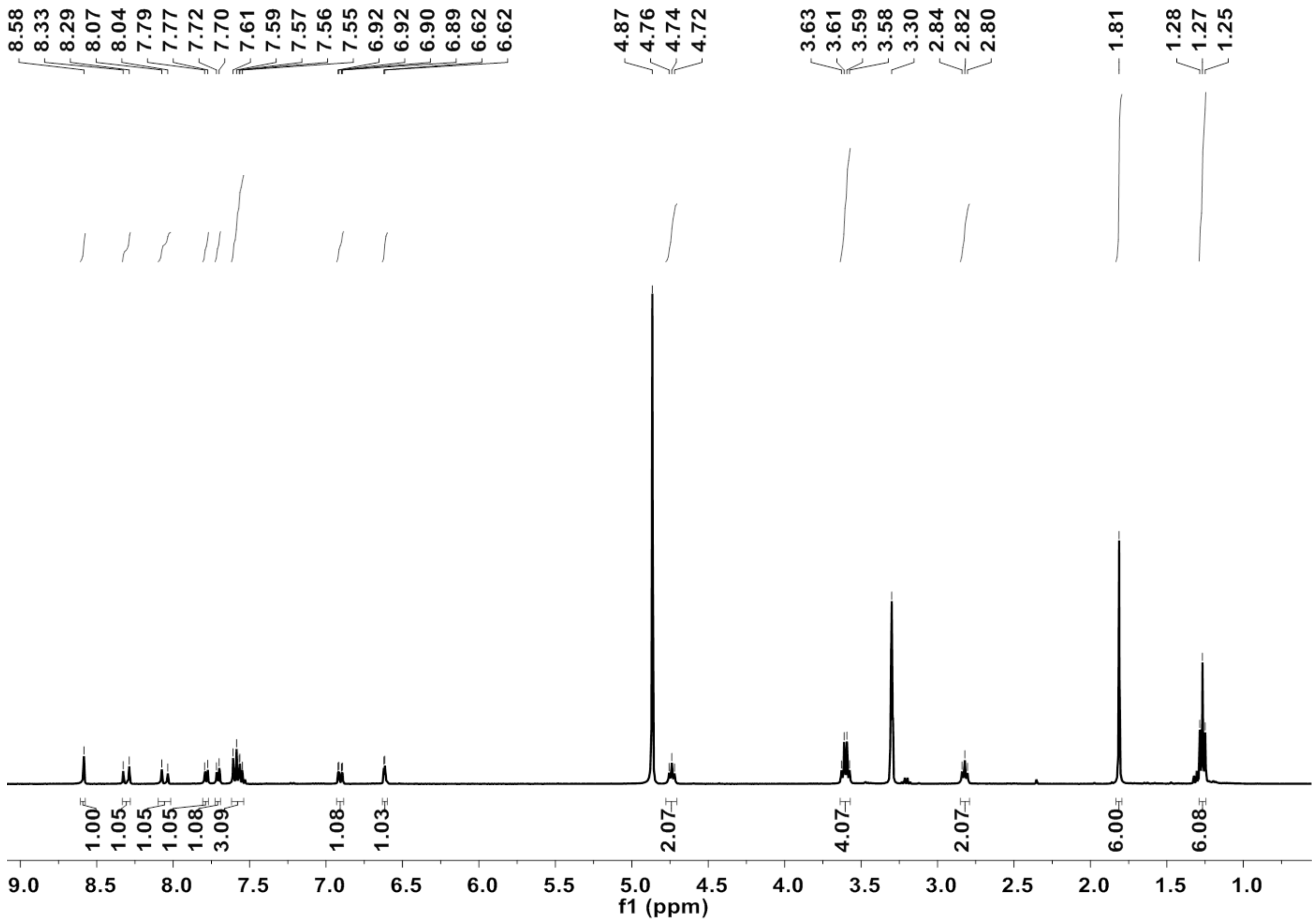

Supplementary Figure 2. ^{1}H NMR of Coupa in CD_3OD.

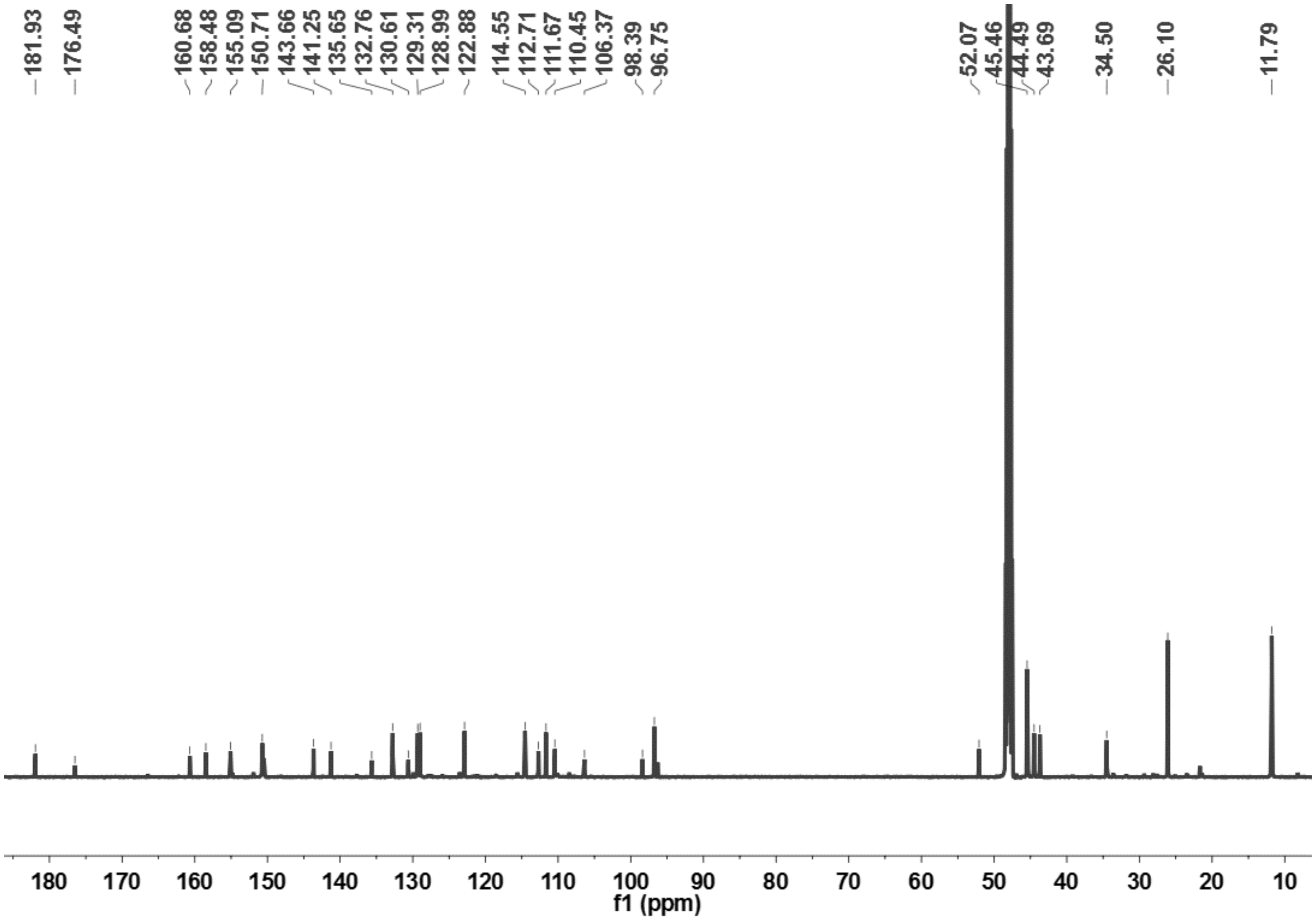

Supplementary Figure 3. ^{13}C NMR of Coupa in CD_3OD.

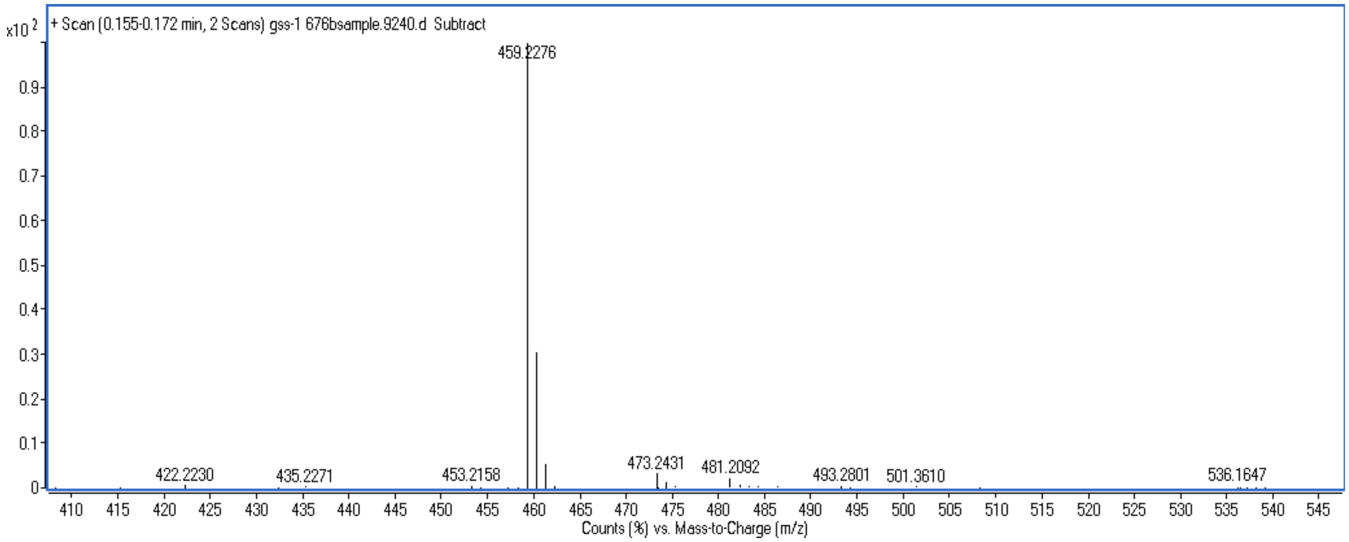

Supplementary Figure 4. High-resolution mass spectrum of Coupa.

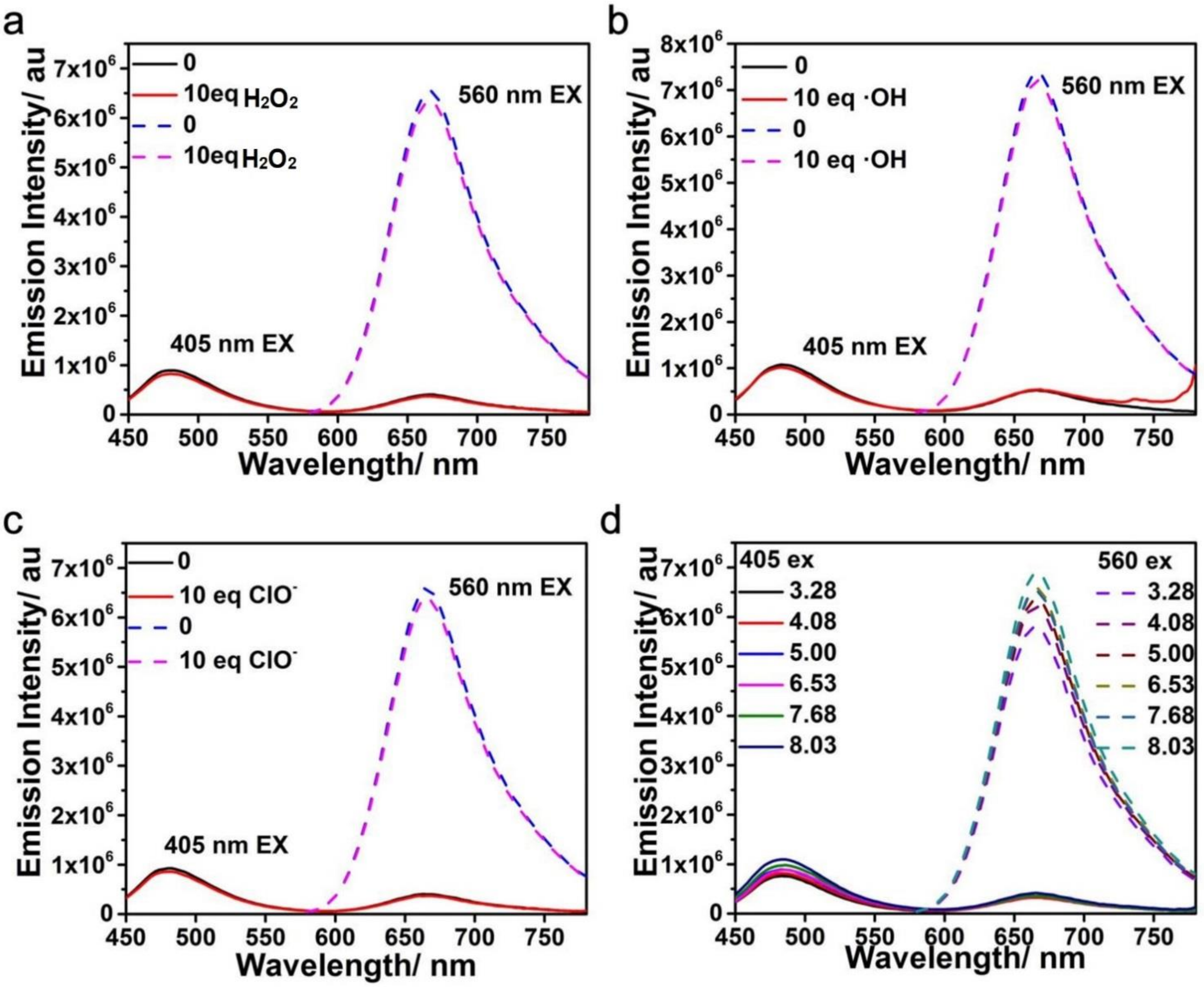

Supplementary Figure 5. Fluorescence spectra of Coupa determined under different conditions. Coupa (10 μM) were mixed with 0 or 10 eq (**a**) H_2O_2, (**b**) ClO^-, and (**c**) •OH or adjusted to different pH values (**d**) for spectroscopic determination upon excitation at 405 nm (solid line) and 560 nm (dashed line) The determination was carried out 10 min after each treatment.

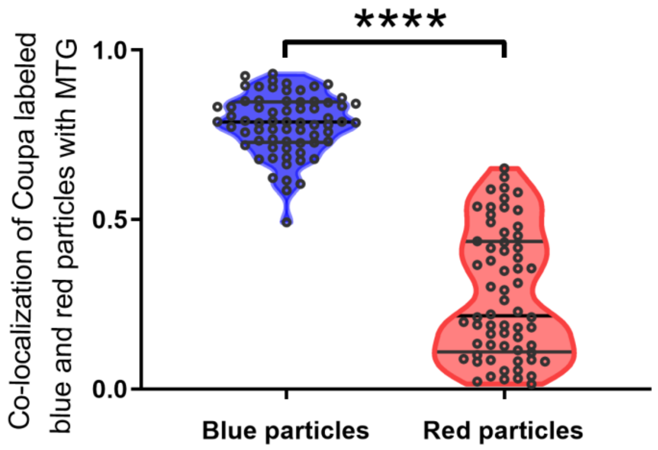

Supplementary Figure 6. Co-localization coefficients of MTG-labeled mitochondria with the Coupa-labeled blue and red fluorescent particles. Data are mean ± SEM (n = 68 areas from 16 cells for blue particles group, and n = 64 areas from 17 cells for red particles). Statistical differences between the two groups were examined by Mann-Whitney test. $P < 0.05$ is considered significant (*$P < 0.05$, **$P < 0.01$, ***$P < 0.001$, ****$P < 0.0001$). Analyzed cells were obtained from three replicates. Source data are provided as a Source data file.

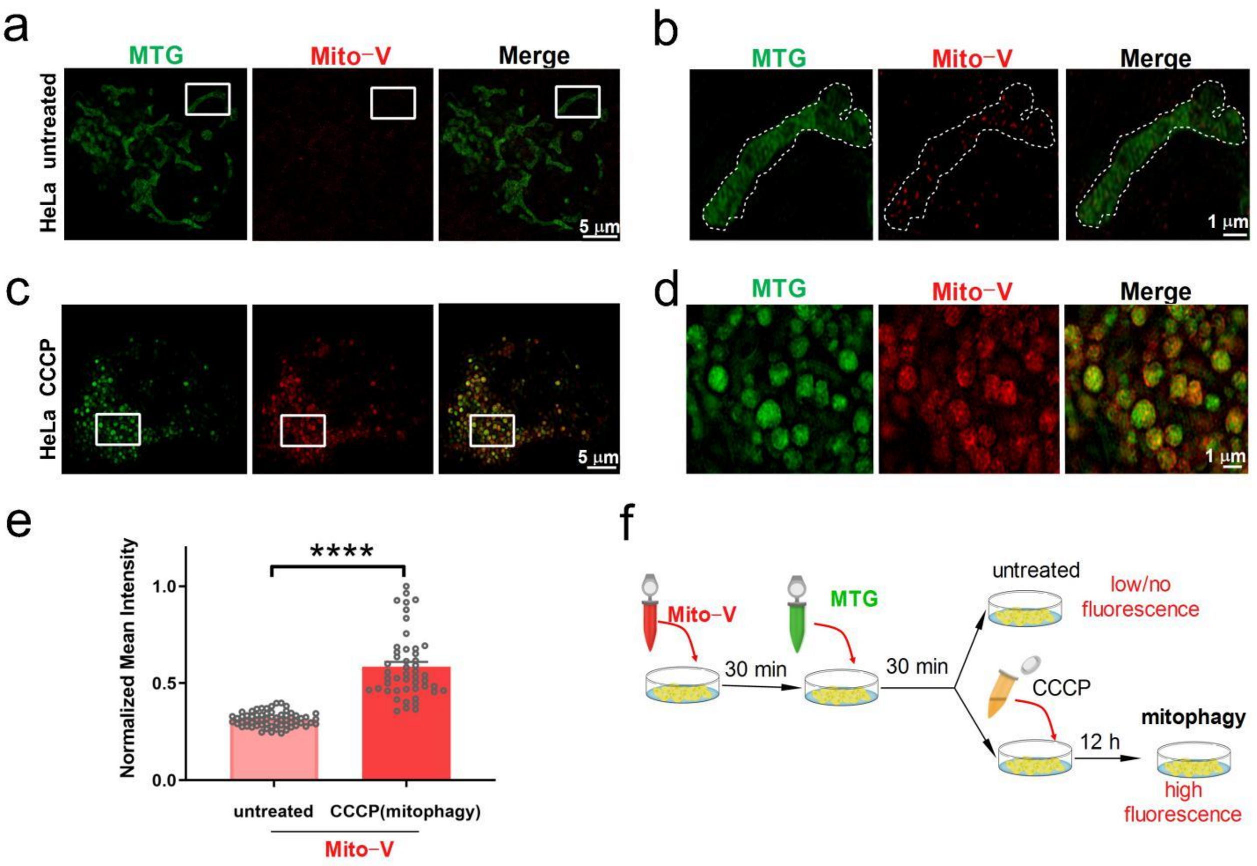

Supplementary Figure 7. SIM imaging of Mito-V-labeled mitochondria in HeLa cells with or without CCCP treatment. (**a**) Mitochondria co-stained with MTG and Mito-V in untreated (**a**) and CCCP-treated HeLa cells (**c**). (**b** and **d**) Zoom-in images of white rectangles in (**a**) and (**c**). (**e**) Normalized mean intensity of Mito-V stained mitochondria with or without CCCP treatment. Data are mean ± SEM (n = 64 areas form 12 cells for untreated group, and n = 48 areas form 16 cells for CCCP group). Statistical differences between the two groups were examined by Mann-Whitney test. $P < 0.05$ is considered significant (*$P < 0.05$, **$P < 0.01$, ***$P < 0.001$, **** $P < 0.0001$). Analyzed cells were obtained from three replicates. (**f**) Schematic representation of Mito-V-labeled mitochondria with or without CCCP treatment. Mito-V imaging conditions: Ex, 561 nm, Em, 570-640 nm; and MTG imaging conditions: Ex, 488 nm, Em, 500 - 550 nm. Source data are provided as a Source data file.

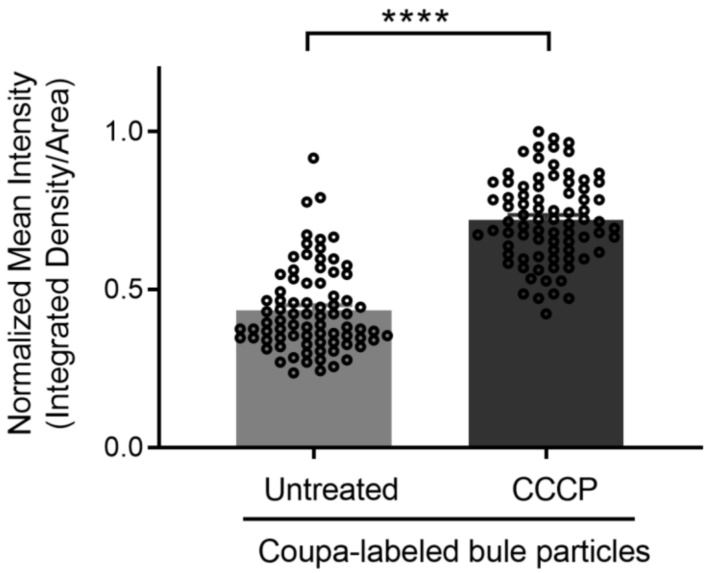

Supplementary Figure 8. Normalized mean fluorescence intensity of Coupa-labeled blue florescent particles in untreated and CCCP-treated HeLa cells. Data are mean ± SEM (n = 80 areas from 20 cells). Statistical differences between the two groups were examined by Mann-Whitney test. $P < 0.05$ is considered significant (*$P < 0.05$, **$P < 0.01$, ***$P < 0.001$, **** $P < 0.0001$). Analyzed cells were obtained from three replicates. Source data are provided as a Source data file.

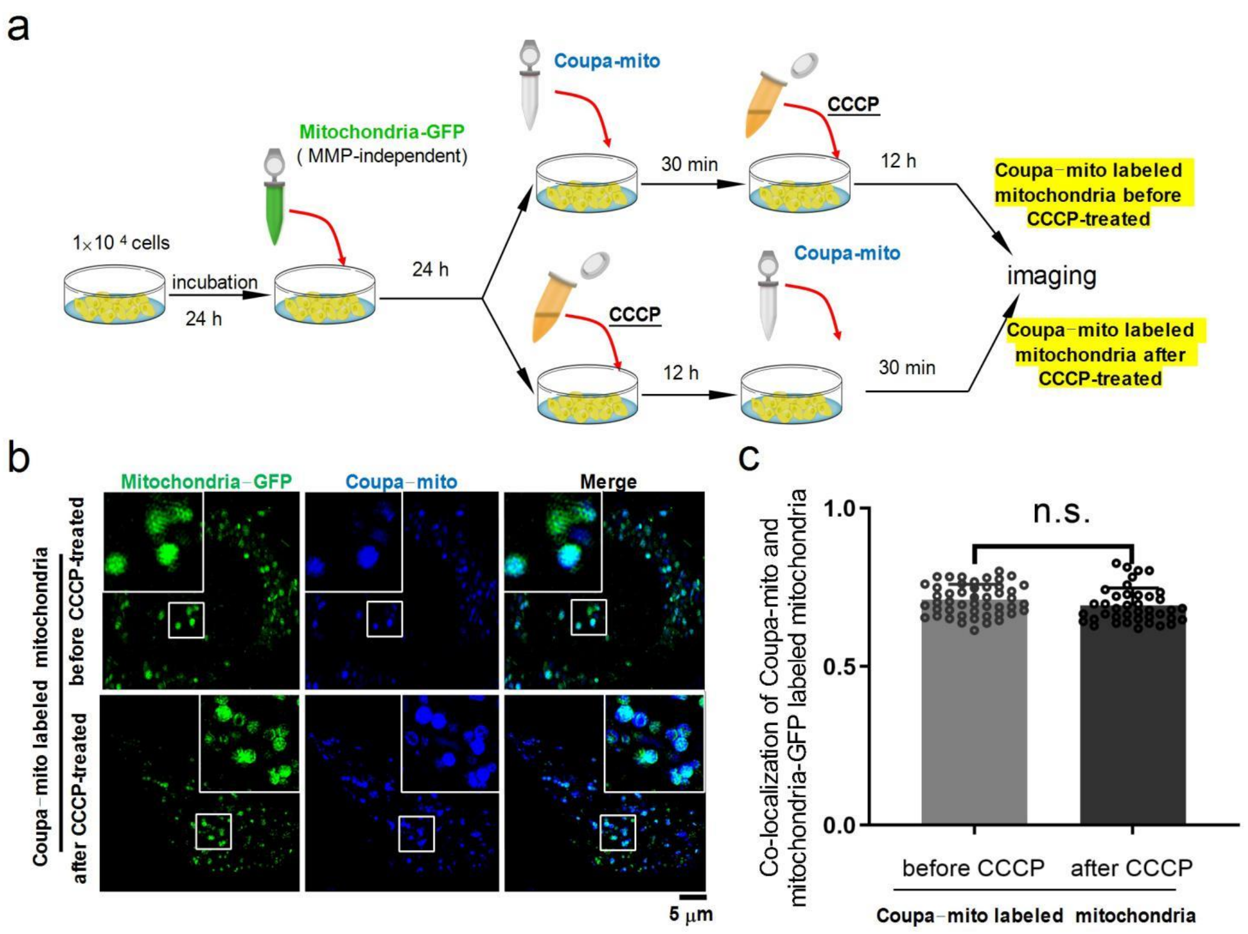

Supplementary Figure 9. SIM imaging of CCCP-treated HeLa cells co-stained by Coupa and GFP via a coupa-mito/GFP dual channel mode. (**a**) Schematic representation of experiments. (**b**) Co-localization imaging of GFP and Coupa (Coupa-mito channel) before or after CCCP treatment, white rectangles indicate Zoom-in images of areas. (**c**) Co-localization coefficients of Coupa-mito with GFP. Data are mean ± SEM (n = 40 areas from 10 cells for each group). Statistical differences between the two groups were examined by Mann-Whitney test. $P < 0.05$ is considered significant (*$P < 0.05$, **$P < 0.01$, ***$P < 0.001$, **** $P < 0.0001$, n.s. for no significant difference). Imaging conditions for Mitochondria-GFP: Ex, 488 nm, and Em, 500-550 nm; Coupa-mito channel: Ex, 405 nm and Em, 420-495 nm. Source data are provided as a Source data file.

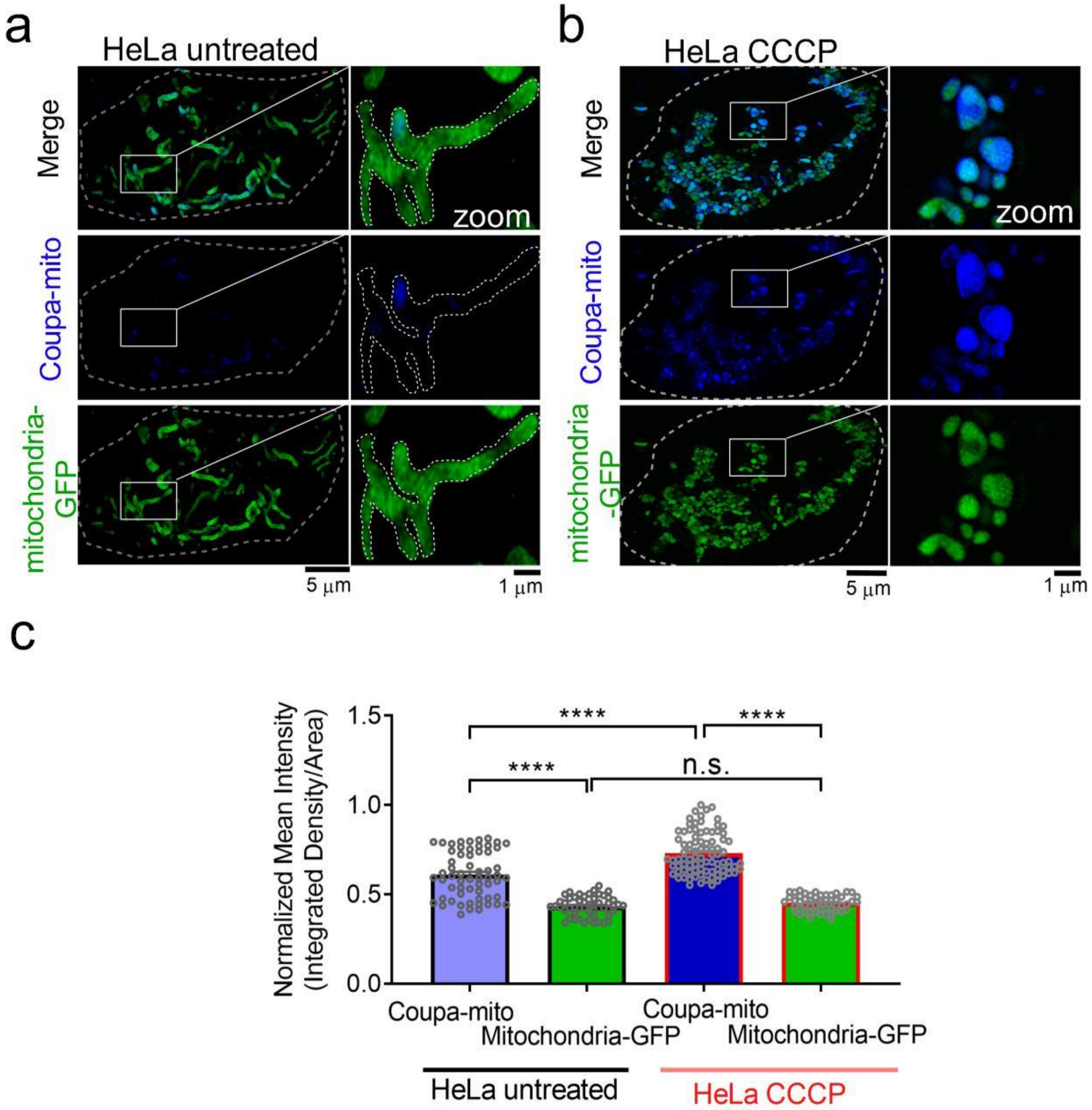

Supplementary Figure 10. SIM imaging of HeLa cells co-stained by Coupa and mitochondria-GFP with or without CCCP treatment. (**a** and **b**) Mitochondria co-stained with Coupa-mito and Mitochondria-GFP in untreated (**a**) and CCCP-treated HeLa cells **(b)**, white rectangles indicate Zoom-in images of region of interests. (**c**) Quantitative analysis of the average fluorescence intensity of Coupa-mito and mitochondria-GFP with or without CCCP treatment. Data are mean ± SEM (n = 60 areas from 15 cells for HeLa untreated-Coupa-mito group, n = 52 areas from 13 cells for HeLa untreated-mitochondria-GFP group, n = 88 areas from 22 cells for HeLa CCCP-Coupa-mito group, and n = 52 areas from 13 cells for HeLa CCCP-Mitochondria-GFP group). Statistical differences between the two groups were examined by Mann-Whitney test and unpaired t test. $P < 0.05$ is considered significant (*$P < 0.05$, **$P < 0.01$, ***$P < 0.001$, ****$P < 0.0001$, n.s. for no significant difference). Mitochondria-GFP channel: Ex, 488 nm, Em, 500 - 550 nm; Coupa-mito channel: Ex, 405 nm, Em, 420-495 nm. Source data are provided as a Source data file.

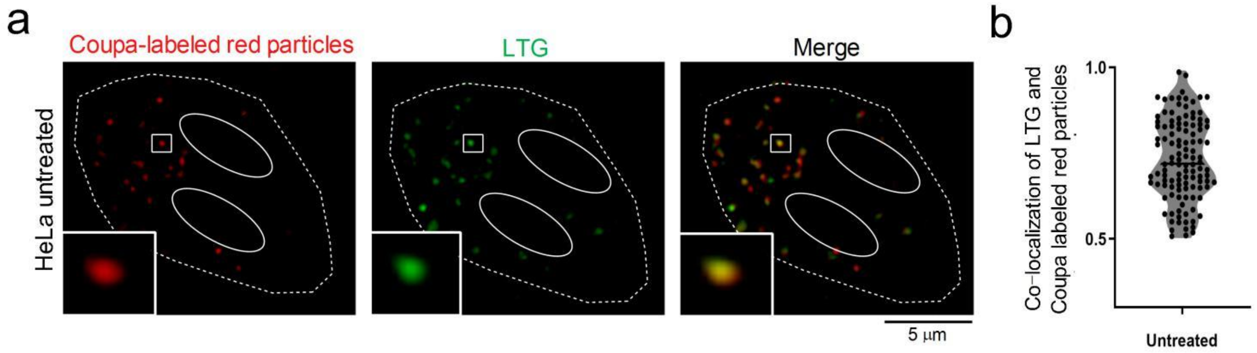

Supplementary Figure 11. The overlap images of Coupa-labeled red particles and lysosome-tracker-green (LTG) labeled lysosomes in untreated HeLa cells (**a**) and co-localization (**b**) with structured illumination microscopy (SIM). Data are mean ± SEM (n = 116 areas from 29 cells). LTG channel: Ex, 488 nm, Em, 500-550 nm; Coupa-lyso channel: Ex, 561 nm, Em = 570-640 nm. Source data are provided as a Source data file.

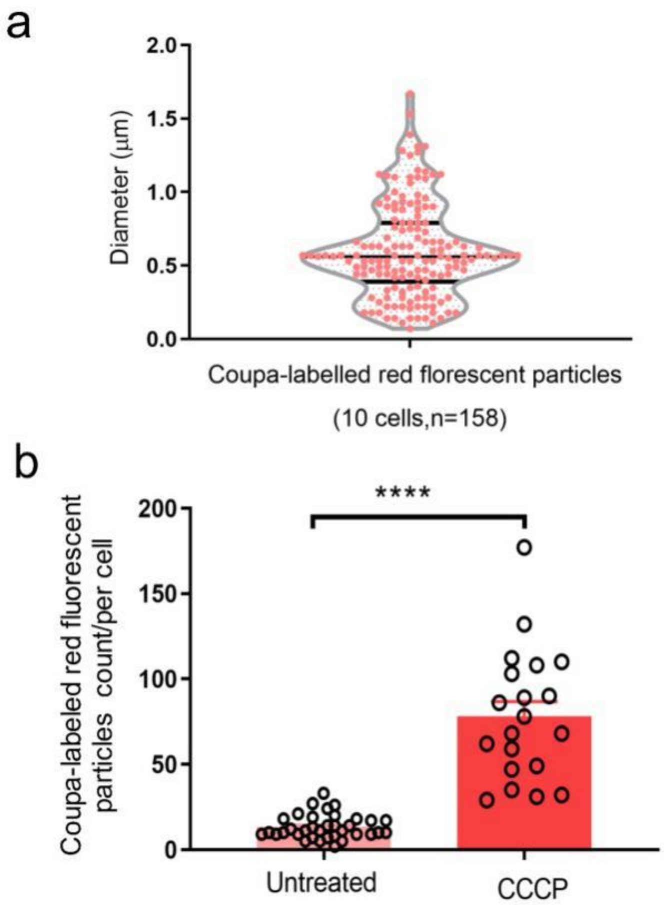

Supplementary Figure 12. Characterization of Coupa-labeled red fluorescent particles in live HeLa cells. (**a**) The diameters of Coupa-labeled red fluorescent particles in HeLa cell. Data are mean ± SEM (n = 158 particles from 10 cells). (**b**) The count of Coupa-labeled red fluorescent particles. Data are mean ± SEM (n = 35 cells for untreated group, and n = 20 cells for CCCP group). Statistical differences between the two groups were examined by Mann-Whitney test. $P < 0.05$ is considered significant (*$P < 0.05$, **$P < 0.01$, ***$P < 0.001$, ****$P < 0.0001$). Source data are provided as a Source data file.

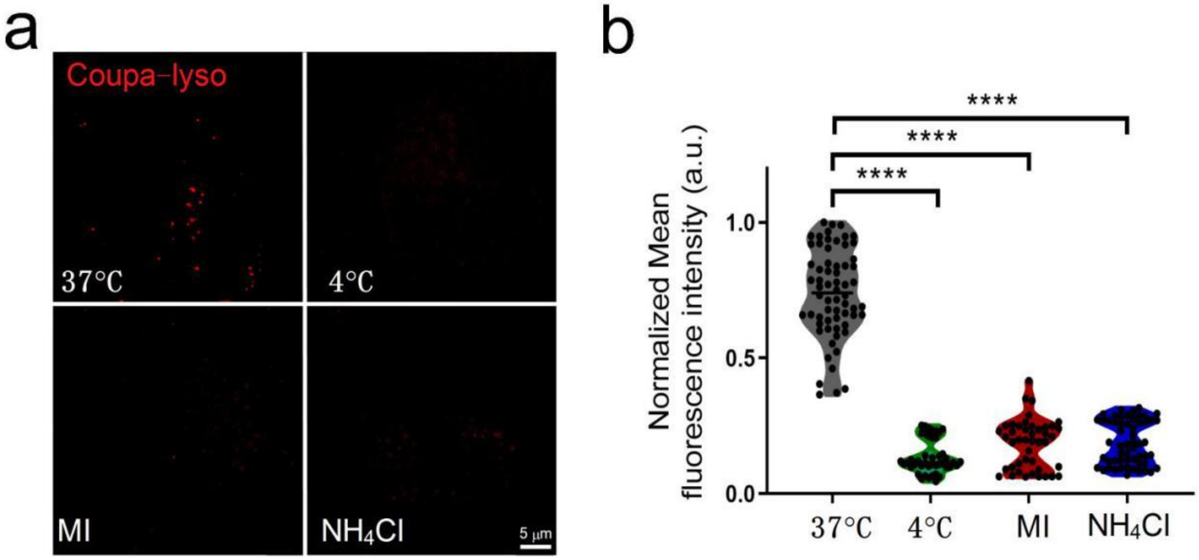

Supplementary Figure 13. Investigation of the cell uptake of Coupa by HeLa cells via SIM imaging. (**a**) SIM images of Coupa-lyso labeled lysosomes at 37°C, 4°C, and with MI or NH_4Cl treatment, and quantitative analysis was shown in (**b**). Data are mean ± SEM (*n* = 64 areas from 16 cells for 37°C group, *n* = 44 areas from 11 cells for 4°C and MI groups, and *n*= 56 areas from 14 cells for NH_4Cl group). Statistical differences between the two groups were examined by Mann-Whitney test. $P < 0.05$ is considered significant (*$P < 0.05$, **$P < 0.01$, ***$P < 0.001$, ****$P < 0.0001$). Coupa-lyso channel: Ex, 561 nm, Em, 570-640 nm. Source data are provided as a Source data file.

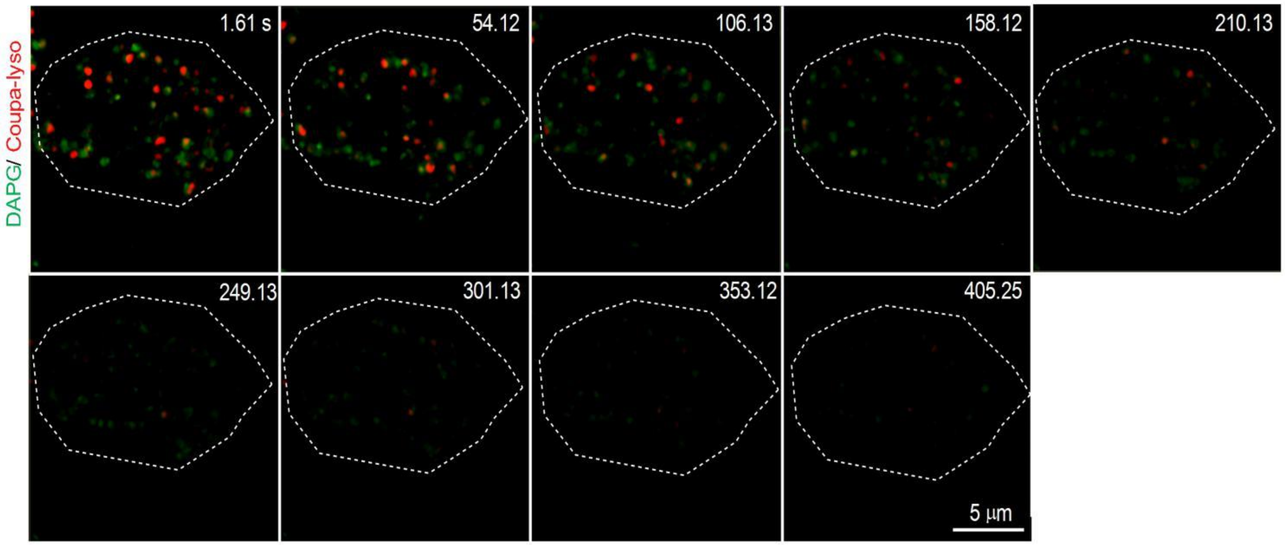

Supplementary Figure 14. Photobleaching comparison of DAPG and Coupa-lyso fluorescence in living HeLa cells. Time-lapse SIM images of CCCP-treated HeLa cells stained with DAPG and Coupa-lyso, showing similar photobleaching properties of DAPG and Coupa-lyso. DAPG channel: Ex, 488 nm, Em, 500 - 550 nm, Coupa-lyso channel: Ex, 561 nm, Em, 570-640 nm.

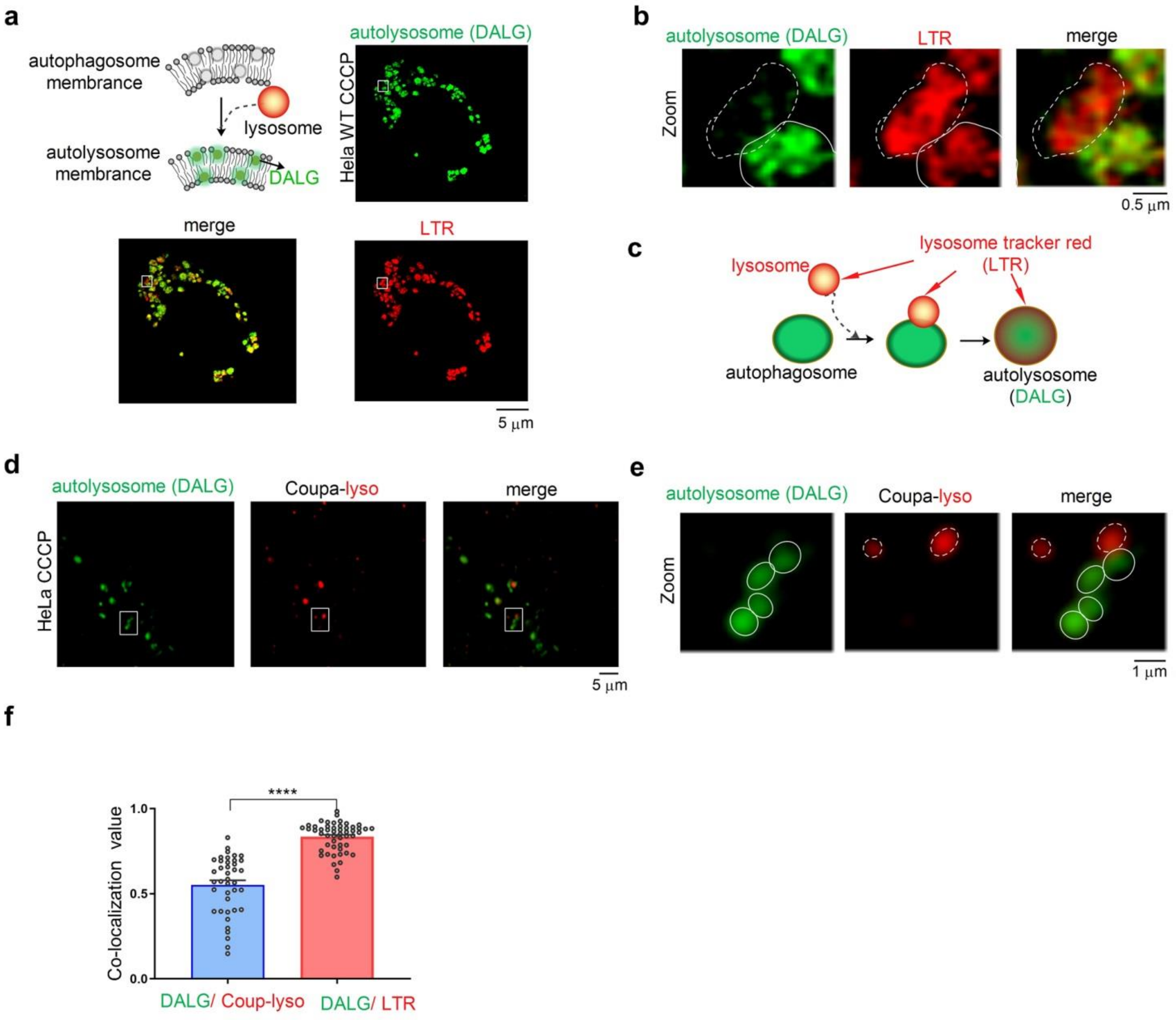

Supplementary Figure 15. Co-localization between lysosomes and autolysosomes in CCCP-treated HeLa cells stained by Coupa and LTR via SIM imaging. (a) Schematic representation and SIM imaging of co-localization of lysosomes stained with commercial lysosome tracker red (LTR) and DALG-stained autolysomes in CCCP-treated HeLa cells. (b) Representative fusion of Coupa-lyso-stained lysosomes and DALG-stained autolysosomes; and (c) schematic representation of commercial LTR unsuitable to distinguish lysosomes from autolysosomes. (d) Co-localization of Coupa-lyso-stained lysosomes and DALG-stained autolysomes in CCCP-treated HeLa cells; white rectangle represents amplification shown in (e). (f) Co-localization coefficients of Coupa-lyso with LTR labeled lysosomes or DALG labeled autolysosomes. Data are mean ± SEM (n = 40 areas from 10 cells for DALG/Coupa-lyso group, and n = 52 areas from13 cells for DALG/LTR group). Statistical differences between the two groups were examined by Mann-Whitney test. $P < 0.05$ is considered significant (*$P < 0.05$, **$P < 0.01$, ***$P < 0.001$, **** $P < 0.0001$). LTR channel: Ex, 561 nm, Em, 570 - 640 nm; DALG channel: Ex, 488 nm, Em, 500-550 nm. Source data are provided as a Source data file.

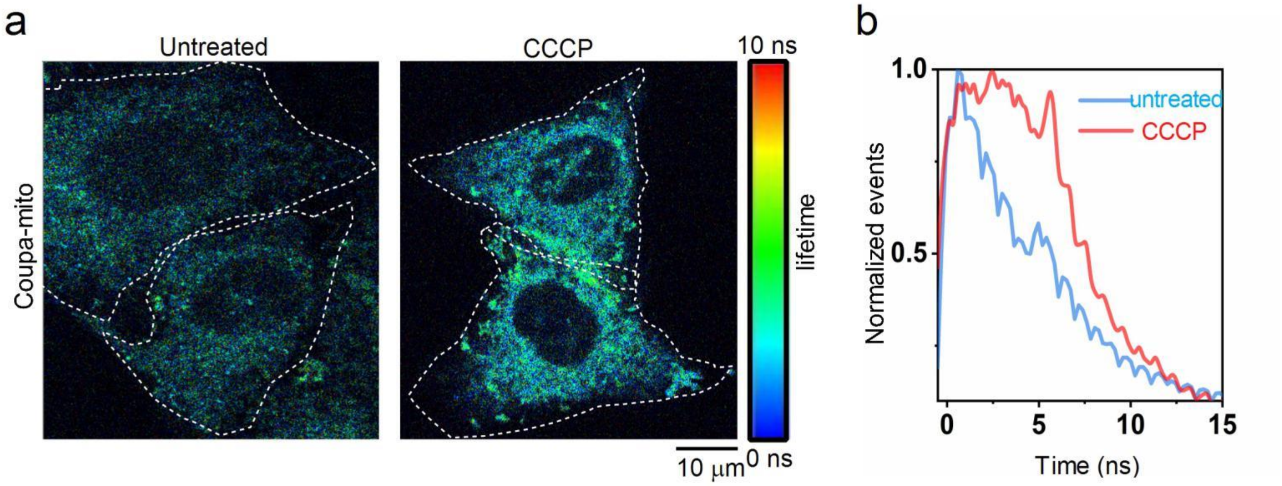

Supplementary Figure 16. Fluorescence lifetime imaging of Coupa-mito labeling mitochondria in live HeLa cells. (**a**) FLIM images of cells with or without CCCP treatment and the related fluorescence decay profiles (**b**). The increase of fluorescence lifetime observed after CCCP treatment also indicated the enhancement of mitochondrial viscosity upon damage. Coupa-mito channel: Ex. 405 nm, Em, 420-495 nm.

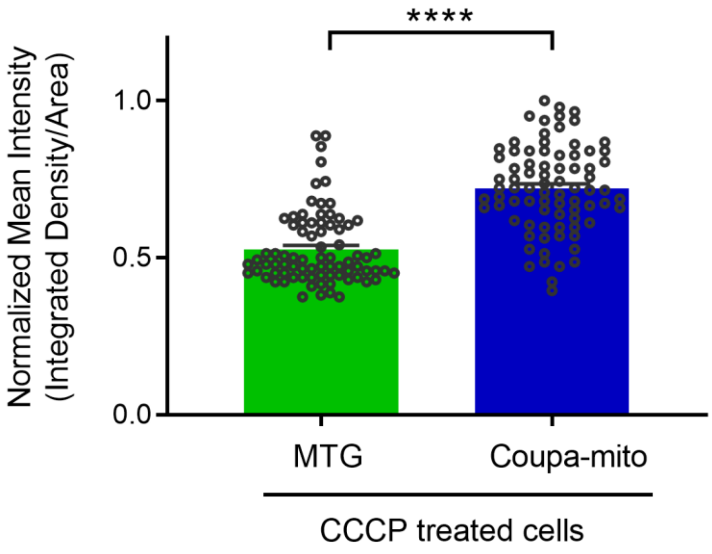

Supplementary Figure 17. The normalized mean intensity of Coupa-mito-stained and MTG-stained mitochondria in CCCP-treated HeLa cells. Data are mean ± SEM (n = 80 areas from 20 cells for each group). Statistical differences between the two groups were examined by unpaired t test. $P < 0.05$ is considered significant (*$P < 0.05$, **$P < 0.01$, ***$P < 0.001$, ****$P < 0.0001$). Source data are provided as a Source data file.

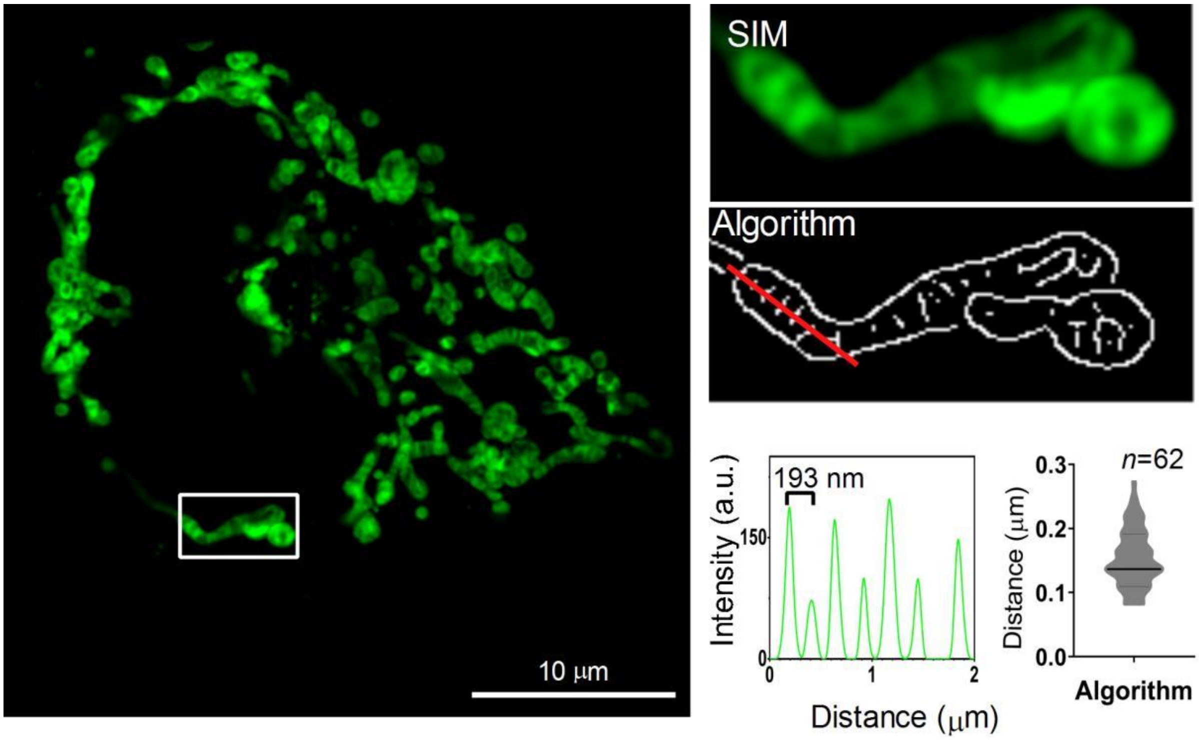

Supplementary Figure 18. The cristae-to-cristae distance determination in HeLa cells via SIM imaging. The mitochondria in untreated cells were stained with MTG, and the raw SIM image was processed using the Canny algorithm to extract the mitochondrial cristae for calculating the cristae-to-cristae distance. Data are mean ± SEM (n = 62 cristae-to-cristae distances). MTG channel: Ex = 488 nm, Em = 500-550 nm. Source data are provided as a Source data file.

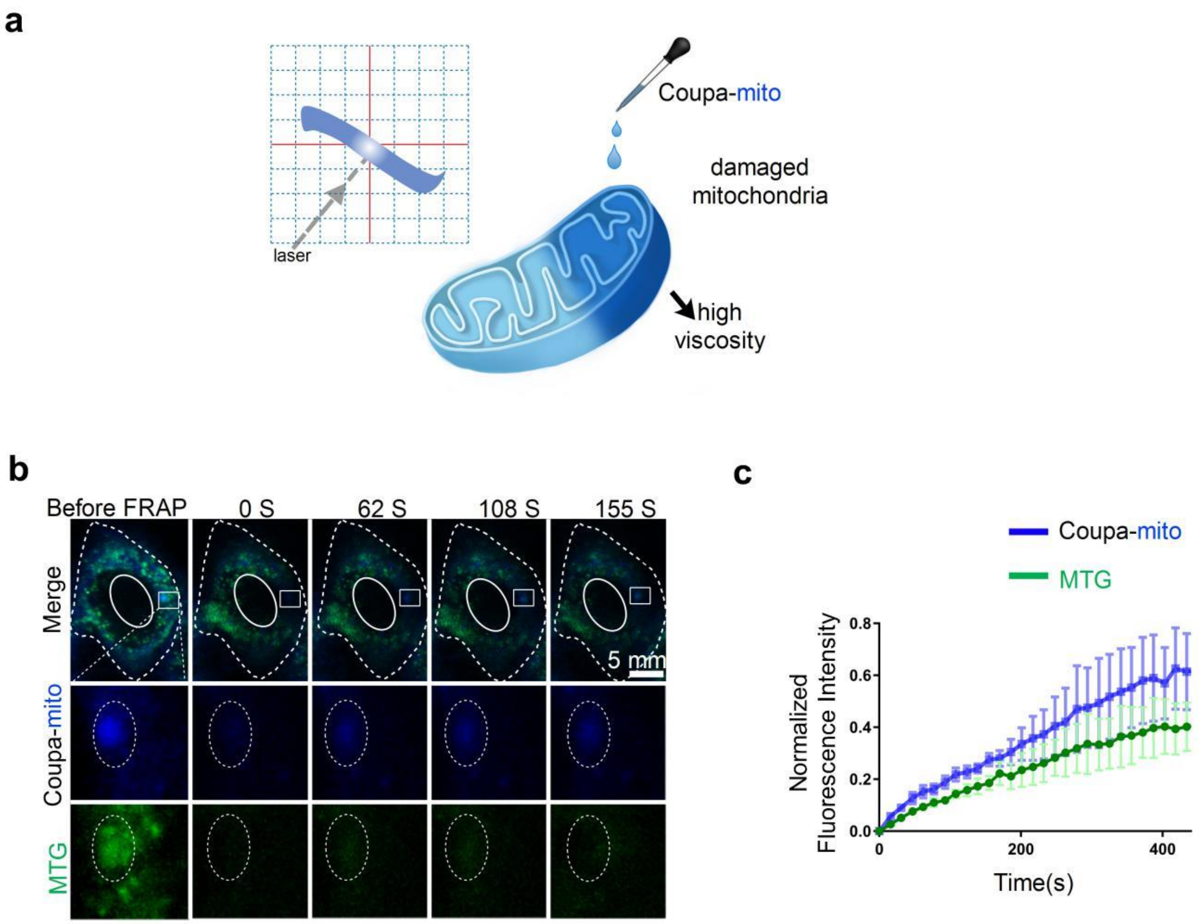

Supplementary Figure 19. Fluorescence recovery after photobleaching (FRAP) assay for Coupa-mito and MTG in HeLa cells stained by both Coupa and MTG. (**a**) Experimental scheme. (**b**) FRAP for mitochondria co-stained with Coupa-mito and MTG in CCCP-treated HeLa cells using a confocal microscope, and (**c**) the normalized fluorescence recovery profile recording during the fluorescence recovery process. Data are mean ± SEM (n = 3 measurements). Coupa-mito channel: Ex, 405 nm, Em, 420-495 nm; MTG channel: Ex, 488 nm, Em, 500-550 nm.

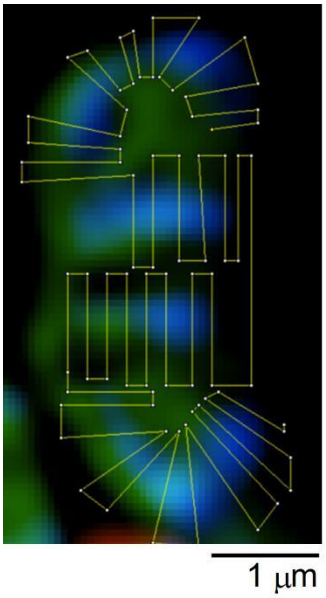

Supplementary Figure 20. The original trajectory for detection the Coupa-mito and MTG fluorescence intensity shown in Figure 4c.
