# Supplementary material for: A dual-labeling probe to track functional mitochondria–lysosome interactions in live cells
Source: Nat Commun. 2020 Dec 8;11:6290. doi: 10.1038/s41467-020-20067-6 (PMC7722883; doi:10.1038/s41467-020-20067-6)
Supplement: Supplementary file 3 — Reporting Summary [file 41467_2020_20067_MOESM3_ESM.pdf]

nature research

Corresponding author(s): Kai Zhang, Weijiang He, Zijian Guo, Jiajie Diao

Last updated by author(s): Oct 26, 2020

Reporting Summary

Nature Research wishes to improve the reproducibility of the work that we publish. This form provides structure for consistency and transparency in reporting. For further information on Nature Research policies, see our Editorial Policies and the Editorial Policy Checklist.

Statistics

For all statistical analyses, confirm that the following items are present in the figure legend, table legend, main text, or Methods section.

n/a	Confirmed	
☐	☒	The exact sample size (*n*) for each experimental group/condition, given as a discrete number and unit of measurement
☐	☒	A statement on whether measurements were taken from distinct samples or whether the same sample was measured repeatedly
☐	☒	The statistical test(s) used AND whether they are one- or two-sided<br>*Only common tests should be described solely by name; describe more complex techniques in the Methods section.*
☐	☒	A description of all covariates tested
☐	☒	A description of any assumptions or corrections, such as tests of normality and adjustment for multiple comparisons
☐	☒	A full description of the statistical parameters including central tendency (e.g. means) or other basic estimates (e.g. regression coefficient) AND variation (e.g. standard deviation) or associated estimates of uncertainty (e.g. confidence intervals)
☐	☒	For null hypothesis testing, the test statistic (e.g. *F*, *t*, *r*) with confidence intervals, effect sizes, degrees of freedom and *P* value noted<br>*Give P values as exact values whenever suitable.*
☐	☒	For Bayesian analysis, information on the choice of priors and Markov chain Monte Carlo settings
☐	☒	For hierarchical and complex designs, identification of the appropriate level for tests and full reporting of outcomes
☐	☒	Estimates of effect sizes (e.g. Cohen's *d*, Pearson's *r*), indicating how they were calculated

Our web collection on statistics for biologists contains articles on many of the points above.

Software and code

Policy information about availability of computer code

Data collection	Nikon Elements (version AR5.11.00 64bit), LSM-710 confocal microscopy(version Leica TCS SP8 3.5.6).
Data analysis	Statistics and graphing were performed using Prism 8 (GraphPad) software, ZEN software (version 2012 SP1), and Nikon Elements(version AR5.11.00 64bit).All images were assembled using ImageJ (version 1.51j8 ,NIH).

For manuscripts utilizing custom algorithms or software that are central to the research but not yet described in published literature, software must be made available to editors and reviewers. We strongly encourage code deposition in a community repository (e.g. GitHub). See the Nature Research guidelines for submitting code & software for further information.

Data

Policy information about availability of data

All manuscripts must include a data availability statement. This statement should provide the following information, where applicable:

- Accession codes, unique identifiers, or web links for publicly available datasets
- A list of figures that have associated raw data
- A description of any restrictions on data availability

All data are available from the corresponding author on reasonable request. Source data are provided with this paper

Field-specific reporting

Please select the one below that is the best fit for your research. If you are not sure, read the appropriate sections before making your selection.

☒ Life sciences ☐ Behavioural & social sciences ☐ Ecological, evolutionary & environmental sciences

For a reference copy of the document with all sections, see nature.com/documents/nr-reporting-summary-flat.pdf

Life sciences study design

All studies must disclose on these points even when the disclosure is negative.

Sample size	All sample sizes are listed in detail in the figure legends and main text. Samples sizes were determined based on the estimates from preliminary experiments and similar studies (Qin J, et al. Nature Communications, 11, 4471 (2020)) so that reasonable statistical analyses could be conducted. For SIM microscopy experiments, 10-29 cells per condition were imaged. For co-localization analysis and average fluorescence intensity samples, individual fluorescent spots or randomly divided cells into four sub-cellular regions for quantitative analysis.
Data exclusions	No data were excluded from the analyses.
Replication	All data presented were from biological replicates. Each experiment was repeated three times independently with similar results. All attempts at replication were successful.
Randomization	Aside from the strong premise for the proposed research, additional steps were taken to ensure rigor and reproducibility, as follows: 1) scientific questions were addressed using complementary technical approaches to ensure that the findings were robust; 2) for studies involving multiple different experimental conditions in the same cell line at the same growth generation, samples were allocated randomly to experimental group. Each experiment was repeated three times independently with similar results.
Blinding	Whenever possible, experimenters were blinded to condition and examiner to exclude bias. In our experiment, data were acquired using imaging setups that performed the measurement independently of the observer. Image acquisition and data analysis were automated and not subject to human bias. For quantitative analysis results (co-localization analysis and average fluorescence intensity), we asked other researchers who were either involved or not involved in the study to examine blinded samples for biological effects.

Reporting for specific materials, systems and methods

We require information from authors about some types of materials, experimental systems and methods used in many studies. Here, indicate whether each material, system or method listed is relevant to your study. If you are not sure if a list item applies to your research, read the appropriate section before selecting a response.

Materials & experimental systems

n/a	Involved in the study	
☒	☐	Antibodies
☐	☒	Eukaryotic cell lines
☒	☐	Palaeontology and archaeology
☒	☐	Animals and other organisms
☒	☐	Human research participants
☒	☐	Clinical data
☒	☐	Dual use research of concern

Methods

n/a	Involved in the study	
☒	☐	ChIP-seq
☒	☐	Flow cytometry
☒	☐	MRI-based neuroimaging

Eukaryotic cell lines

Policy information about cell lines

Cell line source(s)	HeLa cells (from Carolyn M. Price lab, obtained from American Type Culture Collection, ATCC) were used . Further details are provided in the Methods section (Cell treatment and staining).
Authentication	Cell lines were previously authenticated by cytochrome c oxidase subunit I (COI) and short tandem repeat (STR) testing.
Mycoplasma contamination	Cells lines were tested negative for mycoplasma contamination.
Commonly misidentified lines (See ICLAC register)	No commonly misidentified cell lines were used.
